# 107 Nursing Interventions in the Temperature Management of Acute Burn Patients in the Burn Operating Room

**DOI:** 10.1093/jbcr/irac012.110

**Published:** 2022-03-23

**Authors:** Nicole Polsgrove, Madeline Zieger, David Roggy, Brett C Hartman

**Affiliations:** Richard M. Fairbanks Burn Center, Indianapolis, Indiana; Richard M. Fairbanks Burn Center, Indianapolis, Indiana; Richard M. Fairbanks Burn Unit, Indianapolis, Indiana; Richard M. Fairbanks Burn Center, Indianapolis, Indiana

## Abstract

**Introduction:**

The development of hypothermia in the operating room is a known risk that has been well documented in the literature. The typical surgical patient undergoing general anesthesia experiences a temperature loss of approximately 4°F without warming interventions. Burn patients are at a higher risk for hypothermia due to the greater body surface area exposure and evaporative losses related to their burn injury and length of their operative interventions. The purpose of this review is to determine the average loss of body temperature of the burn surgical patient as it pertains to total body surface area (TBSA) injury and the use of warming interventions.

**Methods:**

A two year retrospective review was performed on acute burn surgical cases in our two dedicated burn operating rooms within our burn center. Data obtained included TBSA of each case, pre and post-procedure patient temperatures, maximum OR room temperature, and use of adjunctive warming interventions. The surgical procedures were categorized by percent TBSA burn of < 10%, 10-20%, 21-40%, and >40%.

**Results:**

We identified 415 cases that were included in this review from 2019 and 2020. As expected, patients with larger TBSA involvement led to a greater temperature decline. As seen in Table 1, forced warm air devices were utilized in 67.2% of cases. In our large Burn OR suite, we utilize a heat panel that is integrated in the ceiling above the OR table. Utilization of these devices is determined by the Burn OR nurse. They are either initiated prior to the start of the case or intra-operatively if the patient’s temperature is declining and intervention is required. Mean operating room temperatures were 80.1°F in all cases with cooler room temperatures in the smallest TBSA group. Our average patient temperature decline was 1.25°F in all cases. However, in the largest TBSA group, the mean temperature loss was 2.68°F which is significantly less than the 4°F loss in general anesthesia procedures without warming interventions.

**Conclusions:**

The use of elevated ambient operative room temperatures along with other warming interventions aid in the maintenance of core body temperature in the burn surgical patient. Having dedicated burn operative nurses with investment in the outcome of the burn surgical patient contributes to the overall safety and the maintenance of temperature homeostatic state.

